# Human biodistribution and internal dosimetry of 4-[ ^18^*F*]fluorobenzyl-dexetimide: a PET radiopharmaceutical for imaging muscarinic acetylcholine receptors in the brain and heart

**DOI:** 10.1186/s13550-020-00641-1

**Published:** 2020-06-12

**Authors:** Cameron D. Pain, Graeme J. O’Keefe, Uwe Ackermann, Vincent Dore, Victor L. Villemagne, Christopher C. Rowe

**Affiliations:** 1 Department of Molecular Imaging and Therapy, Austin Health, Heidelberg, Australia; 2 University of Melbourne, Melbourne, Australia; 3 CSIRO, Heidelberg, Australia

**Keywords:** Radiation dosimetry, 4-[ ^18^*F*]fluorobenzyl dexetimide, Muscarinic cholinergic neuroreceptors, Positron emission tomography

## Abstract

**Background:**

4-[^18^F] fluorobenzyl dexetimide (F-DEX) is the first non-subtype selective fluorine-18 labelled tracer for muscarinic receptors (mAChR) used in humans. A recent first-in-human study found high regional brain uptake with low variation in normal subjects. Disturbance of mAChR has been reported in Alzheimer’s and Parkinson’s disease, schizophrenia and depression and various cardiac diseases. The following work assesses the biodistribution, organ tracer kinetics and radiation dose associated with F-DEX.

**Method:**

Dose calculations were based on activity uptake derived from multiple time point whole body PET CT imaging and the organ-specific dosimetric S-factors derived from the ICRP 133 standard man and woman mathematical phantoms. Effective doses were calculated using the latest ICRP tissue weighting factors.

**Results:**

Serial images and time activity curves demonstrate high brain and left ventricular myocardial uptake (5% and 0.65*%* of injected activity, respectively) with greater retention in brain than myocardium. The mean effective dose was in concordance with other ^18^*F* labelled tracers at 19.70 ± 2.27 μSv/MBq. The largest absorbed doses were in the liver (52.91 ± 1.46 μGy/MBq) and heart wall (43.94 ± 12.88 μGy/MBq) for standard man and the liver (61.66 ± 13.61 μGy/MBq) and lungs (40.93 ± 3.11 μGy/MBq) for standard woman. The absorbed dose to all organs, most notably, the red bone marrow (20.03 ± 2.89 μGy/MBq) was sufficiently low to ensure no toxicity after numerous follow-up procedures.

**Conclusions:**

The radiation dose associated with an administration of F-DEX is comparable to that of other ^18^*F* labelled tracers such as FDG (19.0 μSv/MBq) and lower than tracers used for SPECT imaging of muscarinic receptors (I-DEX 28.5 μSv/MBq). Clinical use would likely result in an effective dose less than 4 *mSv* for the ICRP 133 standard phantoms after dose optimisation allowing justification for numerous follow-up procedures. Recent results from first in-human studies and a comparatively low radiation dose make F-DEX an attractive option for future applications of imaging muscarinic receptors in the brain. Further investigation of the potential of F-DEX for imaging parasympathetic innervation of the heart may be warranted.

## Introduction

The first in-human study of 4-[^18^F] fluorobenzyl dexetimide (F-DEX) has recently shown it to have potential as a robust tool for detecting variations in muscarinic acetylcholine receptors (mAChR) in the brain using hybrid positron emission tomography and computed tomography (PET CT) [1]. However, an accurate analysis of its dosimetric properties must be made to determine its safety for patients. It has been shown that a decline in cholinergic function correlates strongly with the symptoms of Alzheimer’s disease [2], and acetyl cholinesterase inhibitors remain the principle symptomatic treatment for this condition. Current research into mAChR agonists aims to develop more effective symptomatic therapy with less systemic side-effects. Molecular imaging has a significant role to play in non-invasive quantification of mAChR and changes over time. Observing temporal changes of brain function with PET results in the accrual of relatively high, though still low level, radiation doses. This emphasises the necessity for accurate dosimetry of such tracers prior to clinical or research use.

There are a number of previously investigated tracers for imaging mAChR in the brain. Iodine-123 labelled iododexetimide (I-DEX) has proven efficacy for single photon emission computed tomography (SPECT) imaging of muscarinic receptors [3–5]. However, tracers labelled with positron emitting isotopes present a more attractive alternative owing to the capabilities of PET CT systems providing superior image quality at reduced radiation dose. A number of carbon-11-labelled tracers which target the mAChR have been developed and investigated in animal and human studies [6–10]; however, an ^18^*F*-labelled analogue presents a much more attractive alternative for the brain or cardiac imaging due to the short physical half life of ^11^*C* significantly limiting its application.The following work presents measurements of the biodistribution and dosimetry of F-DEX from five subjects (three female, two male) by volumetric analysis of multiple time point PET acquisitions. Organ-specific dosimetric S-factors are derived from the International Commission on Radiological Protection (ICRP) publication 133 phantom data [11] and are used to calculate the absorbed dose to a range of organs from which the whole body effective dose is calculated according to tissue weighting factors from ICRP publication 103 [12]. A comparison between the results obtained in this work and other ^18^*F*-labelled tracers is presented and used to make conclusions on the safety of F-DEX as a tracer. The aim of this study was to calculate the biodistribution and resultant dosimetry of F-DEX in a control sample of the population to produce a conclusive estimate of its associated radiation risk if used for future investigations.

## Methods

### Subjects

Five healthy control subjects were recruited for the F-DEX imaging protocol. Table 1 presents details of all subjects and the F-DEX administration. Subjects were assessed by neuropsychological testing and physical examination. This study was approved by the Austin Health Human Research Ethics Committee, and written consent was obtained from all subjects before the imaging studies. No adverse events related to the study drug were observed or reported by the subjects following the F-DEX scan.

**Table 1:** Subject details

Subject	Sex	Age (*y*)	Height (*m*)	Mass (*kg*)	Administered activity* (*MBq*)
1	F	32	1.65	64.3	243
2	F	23	1.72	67.7	246
3	F	36	1.57	68.1	262
4	M	28	1.96	103.6	245
5	M	31	1.84	74.1	246
Mean ± SD		30±4.3	1.75±0.14	75.6±14.4	248.4±6.89

^*^The radiopharmaceutical was administered intravenously as a 5-ml bolus

### Tracer synthesis and purification

Production of F-DEX was fully automated using the iPhase Flexlab synthesis module. The radiosynthesis of F-DEX was achieved by reductive amination of (S)nordexetimide with 4-[ ^18^*F*]fluorobenzaldehyde. No-carrier-added 4-[ ^18^*F*]fluoride was produced from irradiation of [ ^18^*O*]water. [ ^18^*F*]Fluoride was then eluted to reactor 1 by a mixture of K_2_CO_3_ and kryptofix 2.2.2 in a 1:1 solution of acetonitrile/water. The [ ^18^*F*]fluoride was dried by azeotropic distillation at 90 ^∘^C using 1 mL of dry acetonitrile. A solution of 4-formyl- *N*,*N*,*N*-trimethylbenzenaminium trifluoromethanesulfonate (2.5 mg) in dimethylformamide (DMF) (0.5 ml) was added to the dried [^18^F]fluoride, and reactor 1 was then heated at 120 ^∘^C for 25 min to produce 4-[^18^F]Fluorobenzaldehyde. Using 600 μL of DMF containing 12 μL of acetic acid, the reaction mixture was transferred into reactor 2, which was loaded with *NaBH*_3_*CN* (4.5 mg), (S)nordexetimide (3 mg). Reactor 2 was then heated to 120 ^∘^C for 15 min to form F-DEX by one pot reductive amination between 4-[^18^F]fluorobenzaldehyde and (S)nordexetimide. The reaction mixture was purified by C18 Sep-pak and eluted with 1 mL of acetonitrile followed by high-performance liquid chromatography (HPLC) purification with a Gemini Phenomenex 250 ×10 mm semi-preparative HPLC column using gradient elution technique with ammonium formate/acetonitrile (0% acetonitrile–45% acetonitrile over 45 min) as mobile phase. F-DEX was collected at 48 min into 80 mL of water and reformulated in 10% ethanol/saline using the solid phase extraction technique. The reformulated F-DEX solution was then passed through a 0.22 μm filter and recovered in a sterile vial. The total synthesis time was 140 min.

### PET CT protocol

A Philips Ingenuity TF-128 PET CT system was used to acquire five sets of whole body PET and CT images at post-injection time points of 0, 20, 60, 100 and 190 min with each image acquired from the top of the head to the mid-thigh, and each PET image acquired for 60 s per bed position with a total scan time of 10 min. Additional PET and CT brain acquisitions were performed at 120 min post administration for assessment of image quality. All subjects were instructed to void their bladder before entering the imaging room after which, they were positioned arms down supine. Whole body CT images were initially acquired followed immediately by an intravenous administration of F-DEX at the commencement of PET imaging. Each subject remained on the bed for the initial 30 min allowing for a single whole body CT image applicable to the first two PET images. Subsequent PET images were preceded by a whole body CT image. All whole body CT images were acquired at an X-ray tube voltage of 120 kV and a mean exposure of 22.8 mAs (range, 14–30 mAs) providing adequate image quality for delineating dosimetric source regions and providing attenuation correction *μ*-maps for PET images. The additional brain acquisition at 120 min post administration consisted of a low-dose head CT at an X-ray tube voltage of 80 kV and exposure of 30 mAs followed by a single bed position 20 min PET acquisition. Four of the five subjects voided their bladder in the 20 to 60-min and 100 to 190-min intervals, with the remaining subject voiding in the 60 to 100-min and 100 to 190-min intervals. PET data was reconstructed with the Philips BLOB-OS-TF reconstruction algorithm, CT attenuation correction, simulated scatter correction, randoms estimates and dead time corrections.

### Image analysis for tracer uptake

The subjects PET images were coregistered with their corresponding CT images in the quantitative image analysis environment Pmod for the purposes of defining a volume of interest (VOI) over each source organ. The fourteen source organs considered in the dosimetric analysis are the brain, heart contents, kidneys, large intestine, liver, lungs, muscle tissue, red marrow, small intestine, spleen, stomach, thyroid, urinary bladder and remaining tissue. The left ventricular myocardium was also segmented to assess whether there is retention relative to blood pool in the heart chambers but was included in the definition of heart contents for the dosimetric analysis. The average activity uptake in a source organ was determined from the volume weighted activity concentration of the corresponding VOI for eleven of the fourteen source organs with the exceptions being the red marrow, muscle tissue and remaining tissue. Activity in the red marrow was inferred from the average activity concentration inside the vertebrae of the lumbar spine, assuming a red marrow density of 1 g/c*m*^3^ and considering the total mass of red marrow for ICRP 133 standard man and woman. Uptake in the muscle tissue and remaining tissue was determined by defining a VOI over the entire body at each time point, subtracting the activity measured in each of the other VOI to determine a residual activity and assigning the fraction of residual activity to the muscle tissue or remainder tissue according to their mass. The blood clearance rate was estimated by segmenting the aortic arch and determining the normalised activity concentration in the delineated volume. The mean and standard deviation of uptake values in the organs considered were determined from all five subjects at each time point to determine the pharmacokinetic properties of F-DEX.

### Activity uptake models

For all VOI other than the bladder, the activity uptake as a function of time was fitted with a least squares regression to either a sum of two exponentials

1 $$ A(t) = C_{0} e^{-\alpha t} + C_{1} e^{-\beta t}  $$

or a delayed uptake and washout model

2 $$ A(t) = C_{0}\big(1-e^{-\alpha t}\big)e^{-\lambda_{\text{phys}}t} + C_{1} e^{-\beta t}  $$

where for both models *A*(*t*) describes the total activity *A* in the VOI at time *t*, $\lambda _{\text {phys}} = \frac {ln(2)}{t_{1/2}}$ where *t*_1/2_ is the half life of ^18^*F* and *C*_0_, *C*_1_, *α*, *β* are free parameters determined by the regression. Activity in the bladder was assumed to accumulate as

3 $$ A(t) = A_{0,\text{full}}\big(1 - e^{-\lambda_{\text{fill}} t}\big)e^{-\lambda_{\text{phys}}t}  $$

where *A*_0,full_ represents the activity at *t*=0 of a full bladder and *λ*_fill_ is a constant describing the rate at which the bladder fills. The concentration of tracer in the urine was assumed to be constant. Upon voiding, the model assumed instantaneous and total emptying with instantaneous refilling. The activity inside each source organ was assumed to decay according to the physical half life of ^18^*F* after the last time point to ensure the most conservative estimate to the dosimetric calculations.

### Dosimetry

Internal dosimetry was performed according to the formulation presented in the Committee on Medical Internal Radiation Dose pamphlet 21 [13] which specifies the time dependent absorbed dose to a target organ as

4 $$ D(r_{t}, T) = \sum_{r_{s}} \tilde{A}(r_{s},T) S(r_{t} \leftarrow r_{s})  $$

where the term $\tilde {A}(r_{s},T)$ is the cumulative activity inside source organ *r*_*s*_ after time *T* and *S*(*r*_*t*_←*r*_*s*_) is the S factor describing the absorbed dose delivered to the target organ *r*_*t*_ per unit of cumulative activity in source organ *r*_*s*_. S factors for ^18^*F* were derived for standard man and standard woman from the specific absorbed fraction (SAF) data complementing ICRP publication 133. The photon contributions were calculated by linear interpolation of the ICRP 133 photon SAF data to 511 *keV*, and the beta contributions were calculated by numerically integrating the ICRP 133 electron SAF data over an integral normalised probability distribution of the ^18^*F* beta spectrum.

## Results

### Organ uptake

**Fig. 1:**
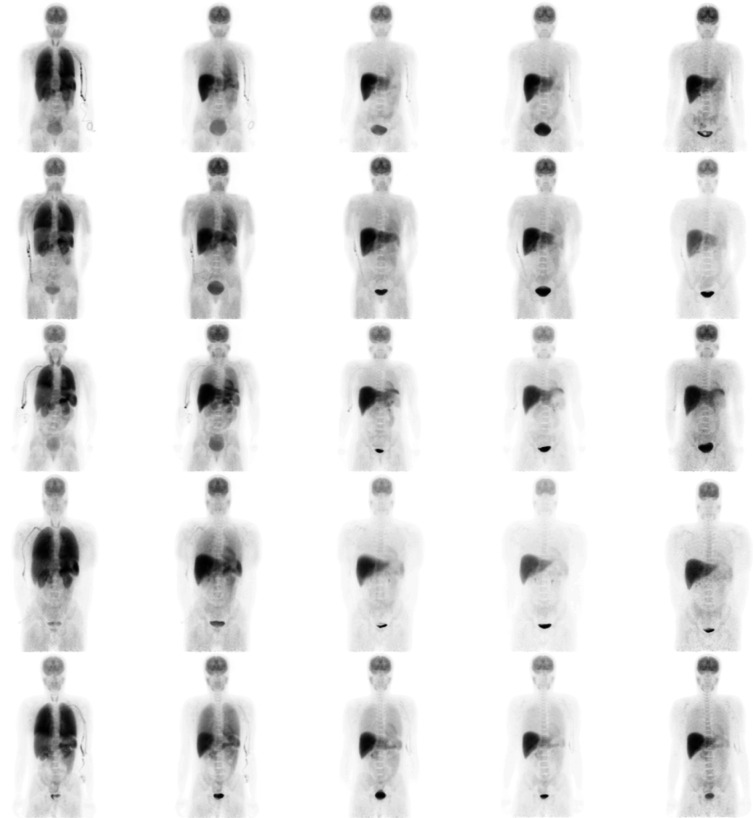
Coronal projections of F-DEX imaging. Acquisitions at time points *t*=0,20,60,100,190 min post administration of F-DEX for each of the five subjects studied. Each row displays one subject and each column represents one time point post administration

**Fig. 2:**
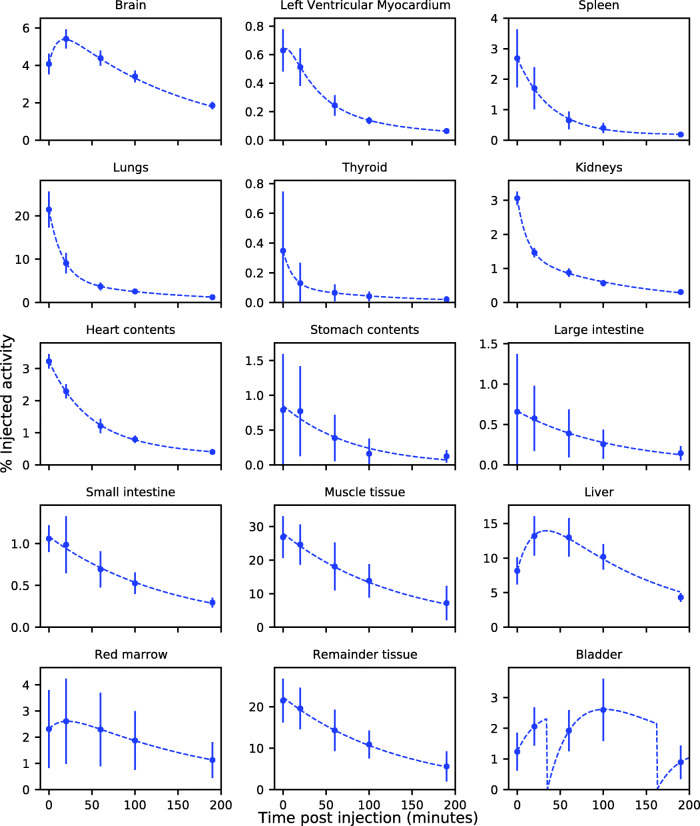
Time-activity curves. The percentage of the initially administered activity as a function of time post injection. Each datum represent the sample mean and sample 95% CI of all five subjects

**Fig. 3:**
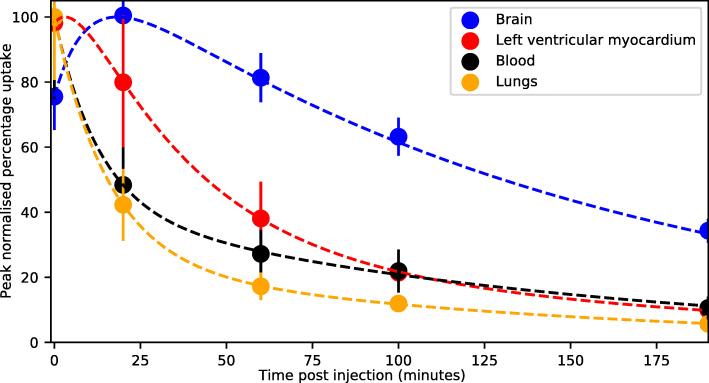
Blood clearance comparison. A comparison of tracer clearance from the brain, left ventricular myocardium, lungs and blood indicates optimal brain imaging likely after 60 min post administration and suggests potential for myocardial imaging between 20 to 60 min post administration

**Fig. 4:**
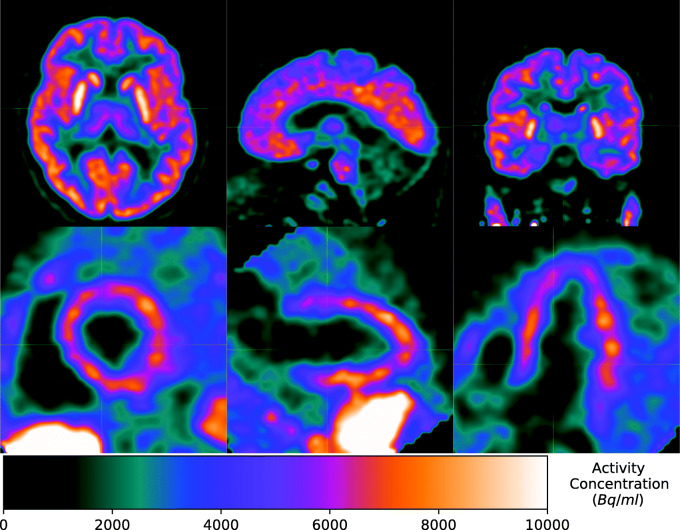
2 mm brain slices and 4 mm heart slices 120 and 60 min post administration. Good contrast is observed in the brain after 120 min and in the left ventricular myocardium after 60 min

Figure 1 shows coronal projections of PET images taken at each time point for each subject studied. It is visually apparent that the high uptake organs include the liver, brain, lungs and urinary bladder (Fig. 1). Uptake in myocardium is also apparent in early images (Fig. 1). Table 2 lists each segmented organ, the equation number of the least squares fitted activity uptake model, the associated fitting parameters for the averaged activity uptake models and the cumulative activity normalised by the administered activity. Figure 2 shows plots of the activity uptake models for all organs listed in Table 2 except peak normalised blood clearance with data points and error bars indicative of the sample mean and sample 95% confidence interval (95% CI) in the VOI activity across all five subjects. The largest cumulative activities were calculated for the muscle tissue, remaining tissue, liver, lungs and brain. The time activity curves in Fig. 2 show fast excretion from the lungs, spleen and kidneys with persistent retention in the brain and liver. Myocardial clearance is much more rapid than brain with little retention at 100 min, but it is slower than lung and blood clearance as shown in Fig. 3. Figure 4 shows 4 mm slices through the heart and 2 mm slices through the brain at time points of 60 and 120 min post administration, respectively. The brain demonstrates excellent uptake in the striatum, significantly less uptake in the thalamus relative to the striatum and negligible uptake in the cerebellum which is consistent with the known distribution of mAChR in the brain. Good contrast is observed between the chambers of the heart and the left ventricular myocardium.

**Table 2:** Biokinetic modelling parameters for delineated regions

Region	Activity uptake model ^*a*^	Fit parameters ^*b*^				Cumulative activity
		*C*_0_ (%)	*C*_1_ (%)	*α* (mi*n*^−1^)	*β* (mi*n*^−1^)	(MBq h/MBq)
Spleen	1	2.517	0.179	0.02624	0.00017	0.02659
Lungs	1	16.064	5.376	0.06423	0.00772	0.16487
Thyroid	1	0.239	0.109	0.09056	0.00909	0.00266
Kidneys	1	1.660	1.400	0.08843	0.00839	0.03360
Heart contents	1	2.470	0.763	0.02277	0.00377	0.04591
Stomach contents	1	0.881	0.0	0.01292	0.0	0.01354
Large intestine	1	0.665	0.0	0.00879	0.0	0.01408
Small intestine	1	1.084	0.0	0.00702	0.0	0.02680
Muscle tissue	1	26.771	0.0	0.00692	0.0	0.65455
Remaining tissue	1	23.314	0.0	0.00708	0.0	0.56611
Blood clearance ^*cd*^	1	58.62	41.39	0.07824	0.00690	N/A
Myocardium (LV) ^*d*^	2	0.201	0.630	0.13425	0.02971	0.00872
Red marrow	2	0.983	2.439	0.05047	0.00532	0.09511
Brain	2	2.485	4.072	0.09423	0.00715	0.16301
Liver	2	26.814	8.176	0.01704	0.01360	0.42313
Bladder model		*A*_0,full_ (*%*)	*λ*_fill_ (*min*^−1^)	void time 1	void time 2	
				(min)	(min)	
Bladder	3	6.3034	0.02329	34.92	162.36	0.10400

^a^Numbering refers to the equation number in the text

^b^The parameters specified are applicable over the domain of 0≤*t*≤190 min

^c^Blood clearance refers to a peak normalised percentage activity concentration in the blood

^d^Left ventricular (LV) myocardium and blood were incorporated into heart contents and remainder tissue in the dosimetric analysis

### Dosimetry

Dosimetry was performed using each subjects individual activity uptake data to estimate the distribution of absorbed doses to organs and whole body effective doses. The sample mean and sample 95% CI of the whole body effective dose averaged over the five subjects studied was calculated to be 19.70±2.27 μSv/MBq using the standard male and female S factors calculated from ICRP 133 SAF data for male and female subjects, respectively. Table 3 lists the sample means and 95% CI calculated over all subjects, male subjects and female subjects. Averaging the effective doses for ICRP133 standard man and standard woman separately highlights an effective dose approximately 15% larger for the standard female. The organs which receive the largest absorbed dose for standard man are the liver (52.91±1.46 μGy/MBq), heart wall (43.94±12.88 μGy/MBq), lungs (42.14±7.16 μGy/MBq), spleen (36.44±7.41 μGy/MBq) and gallbladder (27.27±0.70 μGy/MBq). The organs which receive the largest absorbed dose for standard woman are the liver (61.66±13.61 μGy/MBq), lungs (40.93±3.11 μGy/MBq), spleen (39.69±8.29 μGy/MBq), heart wall (39.33±5.06 μGy/MBq) and thyroid (38.60±28.72 μGy/MBq), respectively. Table 4 lists the sample mean and sample 95% CI of absorbed doses calculated from each subject’s organ uptake data for each organ assigned an ICRP 103 tissue weighting factor in the ICRP 133 standard male and female phantoms. Dosimetry appears consistent for both standard man and standard woman in all organs except the thyroid. Subject 3 as specified in Table 1 saw a peak thyroid uptake a factor of seven greater than that of other subjects positively skewing the female thyroid dose in Table 4.

**Table 3:** Effective dose estimates

	Effective dose (μSv/MBq)		
	Sample mean	Sample 95% CI	Range
Male	17.82	0.96	17.13, 18.51
Female	20.96	2.97	18.91, 24.66
All subjects	19.70	2.27	17.13, 24.66

Female subject effective doses: 19.30, 18.91, 24.66 μSv/MBq

Male subject effective doses: 17.13, 18.51 μSv/MBq

Effective doses calculated using ICRP 103 tissue weighting factors and each individual subjects biokinetic uptake data

**Table 4:** Organ absorbed doses for ICRP 133 standard man and standard woman

Target organs	Absorbed dose (μGy/MBq)					
	Male (*N* = 2)			Female (*N* = 3)		
Lungs	42.14	±	7.16	40.93	±	3.11
Stomach wall	17.05	±	2.97	17.89	±	4.18
Colon	8.74	±	0.17	9.16	±	1.11
Red marrow	14.68	±	2.67	20.03	±	2.89
Breast	6.95	±	0.56	6.71	±	0.74
Ovaries				11.27	±	1.24
Testes	3.75	±	0.07			
Thyroid	16.95	±	2.11	38.60	±	28.72
Oesophagus	13.49	±	1.89	13.74	±	1.63
Urinary bladder wall	23.23	±	6.37	32.63	±	4.15
Liver	52.91	±	1.46	61.66	±	13.61
Endosteum	7.63	±	1.20	10.56	±	1.37
Skin	3.43	±	0.13	4.00	±	0.66
Brain	27.35	±	5.18	33.17	±	0.44
Salivary glands	4.02	±	0.47	5.41	±	0.46
Adrenals	17.99	±	0.18	22.34	±	3.84
Extrathoracic tissue	5.59	±	0.75	6.66	±	0.63
Gallbladder wall	27.27	±	0.70	29.44	±	5.98
Heart wall	43.94	±	12.88	39.33	±	5.06
Kidneys	27.24	±	0.27	31.01	±	1.88
Lymphatic nodes	9.77	±	0.45	10.45	±	1.27
Muscle	7.91	±	0.10	11.54	±	0.77
Oral mucosa	4.08	±	0.50	5.12	±	0.48
Pancreas	15.44	±	0.13	14.90	±	2.54
Prostate	11.19	±	2.08			
Small intestine	9.82	±	0.74	12.03	±	1.59
Spleen	36.44	±	7.41	39.69	±	8.29
Thymus	9.16	±	1.51	10.08	±	0.89
Uterus/cervix				15.72	±	1.80

Each datum is the sample mean ± sample 95*%CI* of the organ doses taken over all subjects of the same sex

## Discussion

Dose estimates for an administration of F-DEX have been previously based on biokinetic data from animal studies with F-DEX or the biokinetic properties of I-DEX in humans determined from SPECT studies. This study presents the first PET CT-based dosimetric analysis of F-DEX in humans. The dosimetric properties of F-DEX are relatively concordant with those of other ^18^*F* labelled tracers due to the relatively short physical half-life (109.7 min) dominating the effects of biological excretion. A mean effective dose to ICRP 133 phantoms of 19.70± 2.27 μSv/MBq is reported for the cohort studied which is in excellent concordance with the mean effective dose of 19.0 μSv/MBq reported for ^18^*F*-fluorodeoxyglucose (FDG) [14]. The excretion and metabolisation of F-DEX appears similar to that of FDG at a macroscopic level which is causal in the similarity of their dosimetric properties. Table 5 presents a comparison of the effective dose per unit of administered activity for F-DEX and other ^18^*F* -labelled tracers.

**Table 5:** Comparison of effective whole body doses for ^18^*F* tracers found in the literature

Author	Tracer	Effective dose (μSv/MBq)	Number of subjects	Measure of error
This work	^18^*F*-DEX	19.70±2.27	5	95% CI
ICRP publication 106 [14]	^18^*F*-FDG	19.0		
O’Keefe et al. [15]	^18^*F*-BAY94-9172	14.67±1.39	3	SD
Doss et al. [16]	^18^*F*-RGD-K5	17.2±0.6	4	SD
Bottlaender et al. [17]	^18^*F*-fluoro-A-85380	19.4 (17.8,21.8)	3	Range

The models for organ uptake show significant tracer metabolism in the liver resulting in relatively large absorbed doses to both the liver and gallbladder wall. Significant excretion through the renal system is also observed resulting in high activity accumulation in the urinary bladder and relatively large absorbed doses to the kidneys, adrenal glands and urinary bladder wall. The administered dose of 250 MBq produced excellent diagnostic image quality in the brain leaving room for dose optimisation to likely reduce the effective whole body dose from the PET examination to below 4 mSv. In comparison, I-DEX for SPECT imaging of mAChR produces an effective dose of 28.5 μSv/MBq [18]. The absorbed dose to the red bone marrow for a 250 MBq administration of F-DEX is 5.01±0.72 mGy for standard woman (3.67±0.67 for standard man) allowing multiple examinations whilst remaining below the ICRP specified annual threshold for red marrow of 250 mGy [19]. A blood sampling approach to red marrow dosimetry was not used due to its inaccuracy for tracers showing specific uptake in the bones or bone marrow. Blood sampling may have provided a more accurate measurement of the blood clearance rate; however, this was considered negligible relative to the additional burden to subjects and staff. The absorbed dose to the kidneys for the same administration is 7.75±0.47 mGy for standard woman (6.81±0.07 mGy for standard man) which will ensure accumulated dose remains well below the ICRP specified limit of 7 Gy for observable effects [19] in the event of numerous follow-up investigations. Potential improvements in the accuracy of bladder dosimetry from urine sampling were deemed negligible relative to the additional workload required for handling and analysing samples. This study has also demonstrated good uptake of tracer in the myocardium with slower clearance than the blood and lungs suggesting F-DEX may be useful for assessing parasympathetic innervation of the heart.

## Conclusions

The mean effective whole body dose to ICRP 133 standard male and standard female phantoms from an administration of F-DEX was found to be 19.70±2.27 μSv/MBq from a sample of five subjects; two male and three female. The effective dose associated with F-DEX is comparable to other 18F labelled tracers and superior to that of the SPECT mAChR tracer I-DEX, presenting an attractive alternative to imaging mAChR from a radiation protection and image quality perspective.

## Data Availability

• Mathematical phantom data including organ masses and SAF data usedto calculate dose source-target dose contributions are available in theICRP repository as supplementary data complementing ICRP publication133 (https://www.icrp.org/publication.asp?id=ICRP%20Publication%20133) • Imaging datasets from which activity uptake was derived are availablefrom the corresponding author upon reasonable request.

## Abbreviations

F-DEX: 4-[^18^F] fluorobenzyl dexetimide
mAChR: Muscarinic acetylcholine receptors
PET CT: Hybrid positron emission tomography and computed tomography
I-DEX: Iodine-123 labelled iododexetimide
SPECT: Single photon emission computed tomography
ICRP: International Commission on Radiological Protection
DMF: Dimethylformamide
HPLC: High-performance liquid chromatography
VOI: Volume of interest
SAF: Specific absorbed fraction
FDG: ^18^*F*-fluorodeoxyglucose
